# Biogeochemical dynamics in a marine storm demonstrates differences between natural and anthropogenic impacts

**DOI:** 10.1038/s41598-024-59317-8

**Published:** 2024-04-16

**Authors:** Justin Tiano, Rob Witbaard, Theo Gerkema, Karline Soetaert

**Affiliations:** 1 Wageningen Marine Research, Wageningen University & Research, IJmuiden, The Netherlands; 2 Department of Estuarine and Delta Systems, Royal Netherlands Institute for Sea Research (NIOZ), Yerseke, The Netherlands

**Keywords:** Storm impacts, Fisheries impacts, Biogeochemistry, Water column, Element cycles, Environmental impact, Marine chemistry, Fluid dynamics, Physical oceanography

## Abstract

This study explores the impact of a wind storm on sediment resuspension and marine biogeochemical dynamics. Additionally, the storm took place during an expedition researching bottom trawling, enabling the direct comparison of certain natural and fisheries-related disturbances. The storm was initiated by a decline in atmospheric pressure and a 2 h period of gale force winds, which was followed by over 40 h of elevated bottom currents. Storm induced turbidity, potentially a cumulative post-fishing impact, was remarkably higher compared to what was observed in a recent trawling event. Storm-induced mixing and movement of water masses led to decreased silicate and increased phosphate concentrations in the water column, accompanied by lower salinity and higher fluorescence. The erosion depth of the seabed averaged around 0.3 cm during the peak turbidity period. Trawl-induced erosion in the area has been measured at over twice that depth, and has been linked to intermittent reductions in near-bed oxygen levels. In contrast, storm-induced turbidity coincided with increased oxygen due to wave mixing, suggesting inherent differences in how trawling and storms can oxidize reduced substances. These findings suggest that storms have a greater regional impact, whereas the local impacts of bottom trawling on biogeochemistry can be more significant.

## Introduction

Marine primary production has been estimated to vary between 40 and 60 Pg of carbon per year worldwide^1^ and depends greatly on nutrient input and bioavailability^2^. The regulation of nutrient availability is influenced in large part by sediment resuspension, which plays a critical role in modulating nutrient levels through release and adsorption processes^3–6^. While sediment resuspension occurs naturally through processes such as tidal currents and episodic storm events, anthropogenic drivers of sediment resuspension, such as dredging and bottom trawling, are also known for their capacity to directly alter carbon and nutrient dynamics^7–9^. Furthermore, natural disturbances can exacerbate the input of human-induced sources of nutrients and organic matter (OM) by augmenting the influx of land-based contaminants^10,11^. Understanding the complex interplay between natural and anthropogenic-mediated sediment disturbances is imperative for objectively quantifying and managing their impacts on marine biogeochemistry^9,12^.

Episodic storm events and more predictable resuspension events driven by daily tidal current patterns, play critical roles in particle transport and sediment distribution on the seafloor^12,13^. The combined sediment transport resulting from waves and currents, is significantly higher than the sediment transported solely from bottom trawling^13^. Natural processes including enhanced water currents and vertical mixing of the water column during storms, can obscure the detection of anthropogenic disturbances such as bottom trawling^14,15^. Both natural and anthropogenic perturbations have been linked to similar environmental impacts^9,15^.

Sediment resuspension, whether induced by storms or bottom trawling, has been shown to diminish OM quality by accelerating degradation rates through heightened exposure to oxygen^9,16^. However, higher observed levels of degraded OM associated with trawl-induced resuspension suggests that trawling may expose OM and nutrients within comparatively deeper layers of sediment^17^. Disturbance from trawling and storms can increase nutrient concentrations in water column can through enhanced mineralization and desorption processes^5,6,18^. The specific consequences of elevated nutrients is context dependent as it can fulfill an important function for marine primary production but it also increases the risk of hypoxic conditions.

Low oxygen zones can be triggered by an influx of OM leading to enhanced degradation processes. While there is a risk of hypoxia following trawl-induced resuspension, storm-enhanced nutrient input is known to cause rapid phytoplankton blooms contributing to the formation of hypoxic zones upon their degradation^19–21^. The upwelling of nutrient-rich water or enhanced runoff occurring after a storm are potential mechanisms for elevated OM fluxes, with the latter increasing the chances of human-mediated contaminants from entering the system^22,23^.

Particle transport and vertical mixing driven by storm-induced currents can stimulate biogeochemical cycling in the water column after receiving an influx of OM and nutrients^11,20,24,25^. Oxygen-consuming OM mineralization has been documented to be a prominent biogeochemical process taking place in the water column after storm disturbance^11,21,26,27^, although, storms have also been linked with reduced sedimentary OM and lower fluxes of dissolved inorganic carbon from the seafloor^28^. The storm-induced formation of a temporary frontal convergence zone has been observed to potentially trap nutrients within a system as highlighted in a recent study^10^.

## Background

The current study was conducted in a permanent frontal zone located in the southern North Sea in the area known as the Frisian Front. Approximately 60 km from the coast of the Netherlands at 30–40 m depth, the Frisian Front serves as a convergence zone for northern and southern water masses. It is characterized by reduced currents that facilitate the deposition of silt and OM^29–31^. Storm-induced erosion in the Frisian Front is hypothesized to play an important role in exporting sediments, thereby maintaining relatively low net sediment accumulation^30,32^. Furthermore, sediment resuspension from bottom trawling in the Frisian Front is known to cause intermittent reductions in water column oxygen and the release of porewater nutrients^4,7^.

In 2017, an expedition researching bottom trawl impacts in the Frisian Front experienced a summer storm in the final days of the campaign^7,33^. This unexpected weather event presented a rare opportunity to compare and evaluate the impact of bottom trawling versus a storm in the off-shore North Sea. Results presented here focus on the findings from the storm impacted portion of this research expedition which took place after experimental trawling activities^7,33^. The physical and biogeochemical observations obtained before, during and after the storm, provide unique insights on how naturally occurring phenomena, such as a summer storm, can affect marine ecosystem functioning and how these relate to potentially cumulative impacts on bottom disturbance caused by trawling.

## Results

### Atmospheric conditions

**Figure 1:**
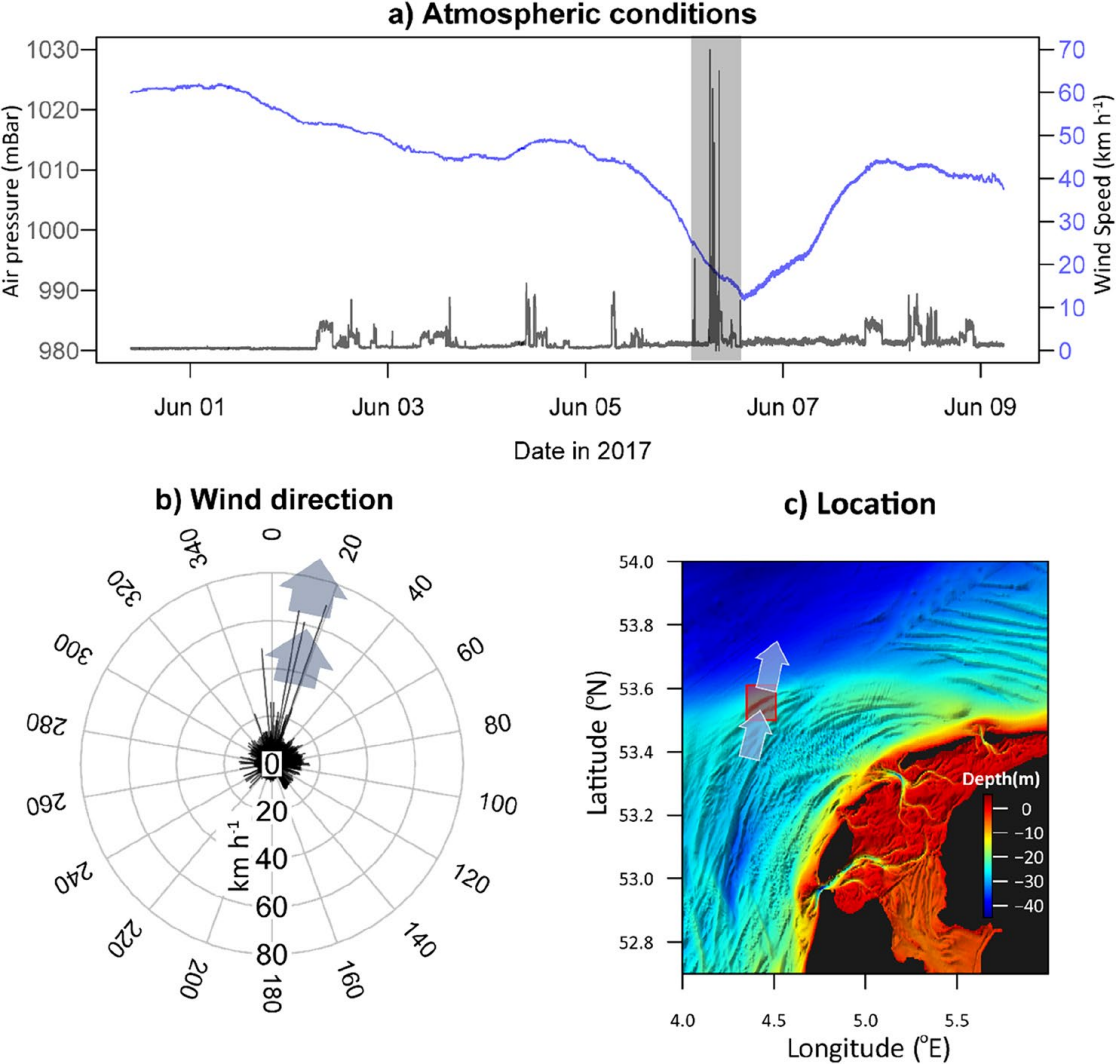
Atmospheric pressure and windspeeds with the shaded region representing the period of high wind gusts (**a**). Wind direction (degrees) and velocity (km h^−1^) with arrows showing the period of high wind gusts (**b**) measured from the RV Pelagia during 1–9 June 2017. Location of the Frisian Front with respect to depth and the shoreline of the Netherlands with arrows showing the wind direction on 6 June 2017 (c). *Traditional meteorological convention gives the wind direction in terms of its origin (i.e. southerlies = coming from the south). Here we chose to communicate wind direction consistently with the traditional water current convention which refers to the direction where the currents are flowing. See the arrows in the plot for clarification.

The barometric pressure gradually decreased from 1025 mBar on 1 June 2017 to 1012 mBar on 5 June 2017. This calm period before the storm displayed relatively low wind speeds with maximum gusts of 15 km h^−1^. On June 6th the air pressure descended rapidly reaching 990 mBar (Fig. 1a). The steep drop in air pressure was accompanied with a rapid veering of the wind direction in the late morning of 6 June to 200° and increasing windspeeds from that direction. Wind gusts during this two-hour period reached a maximum of 70 km h^−1^ (Fig. 1a, 1b). Windy conditions coincided with the experimental bottom trawling which was documented in two studies^7,33^ and within a few hours after the final trawl treatments concluded, a severe summer storm developed causing an unworkable sea state.

### Water movement

**Figure 2:**
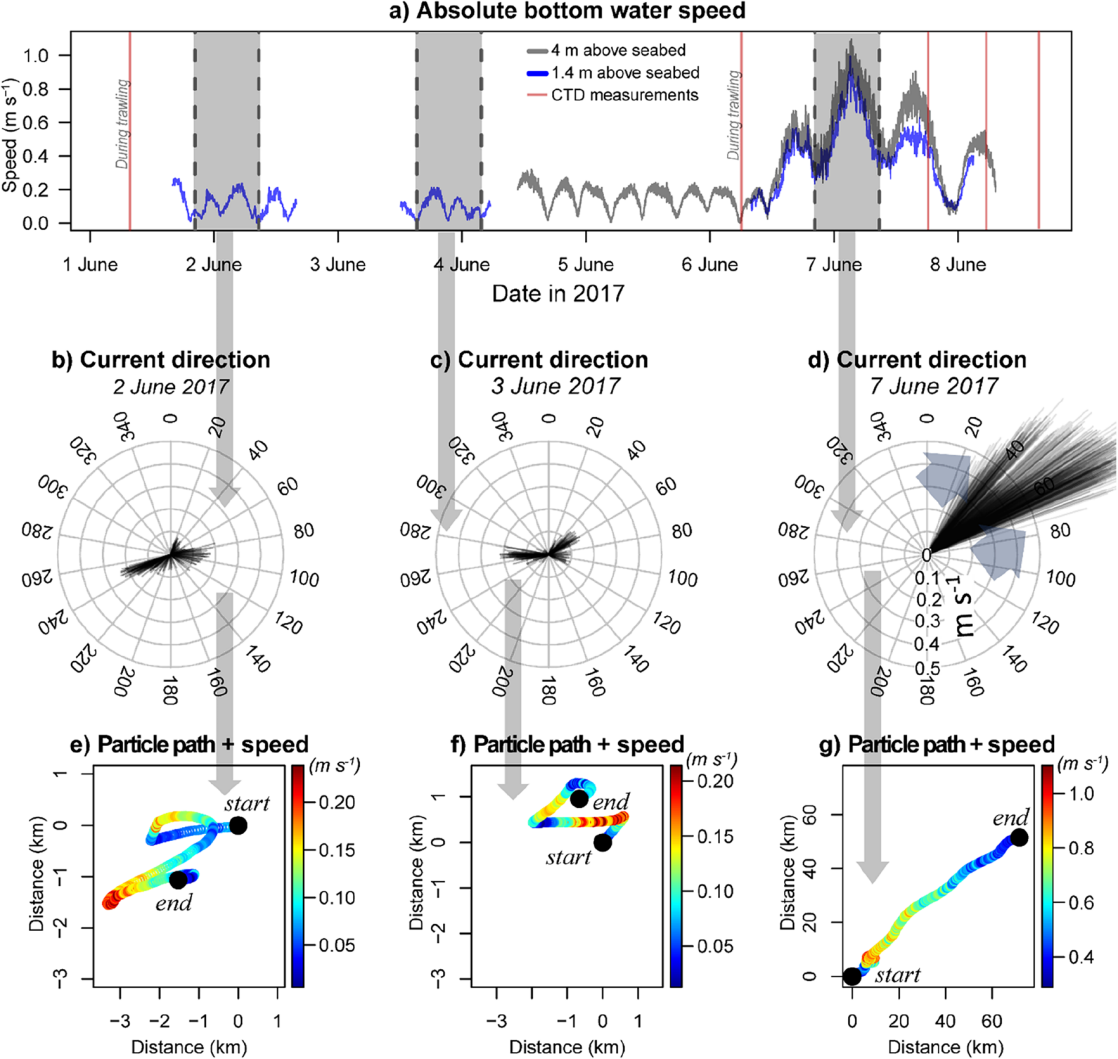
Absolute current speed from 1.4 and 4 m (m) above the seabed with shaded areas delineating specified periods (~ 12.4 h tidal cycle) between high tides (**a**), water current speeds (m s^−1^) and trajectories (degrees) within the selected periods for calm (**b**–**c**) and stormy conditions (**d**). Transport paths (km) calculated by integrating current speeds over time for calm (e–f) and stormy conditions (g).

Figure 2 shows a summary of the measured water currents (absolute current speeds) for the entire period before and during the storm event. The figure shows that the days leading to June 6th show a well-defined tidal cycle with a 12.4 h period and absolute bottom current speeds up to 0.24 m s^−1^ in a predominantly eastward or westward direction depending on the tidal cycle. During this period, the horizontal advection of water masses suggested a maximum residual transport up to 3 km away (Fig. 2e-f), conveying a typical tidal excursion. The following period which featured bottom current speeds above 0.35 m s^-1^ was defined as the ‘storm period’ observed in this study.

The increased windspeeds on the morning of 6 June 2017 were followed by an acceleration of bottom water currents occurring approximately 8 h later. Measurements from 4 m above the seabed show a steep increase in water current speeds from 0.12 to 0.70 m s^−1^ within a few hours in an exclusively north easterly direction (Fig. 2a). Near-bed current speeds reached a maximum of 1.1 m s^−1^ on in the afternoon of 7 June 2017. Mean current velocities from 4 m above the seabed during the storm were, on average, 22% higher than from data collected 1.4 m above the sea bottom (Fig. 2a). Regardless of the phase in the tidal cycle, water trajectories during the storm were unidirectional (Fig. 2g). Estimations of transport distances reached a maximum of 50 km north and 65 km east during the 12.4 h period between high tides at the height of the storm (4.2–5.4 km h^−1^; Fig. 2g).

### Biogeochemical depth profiles

**Figure 3:**
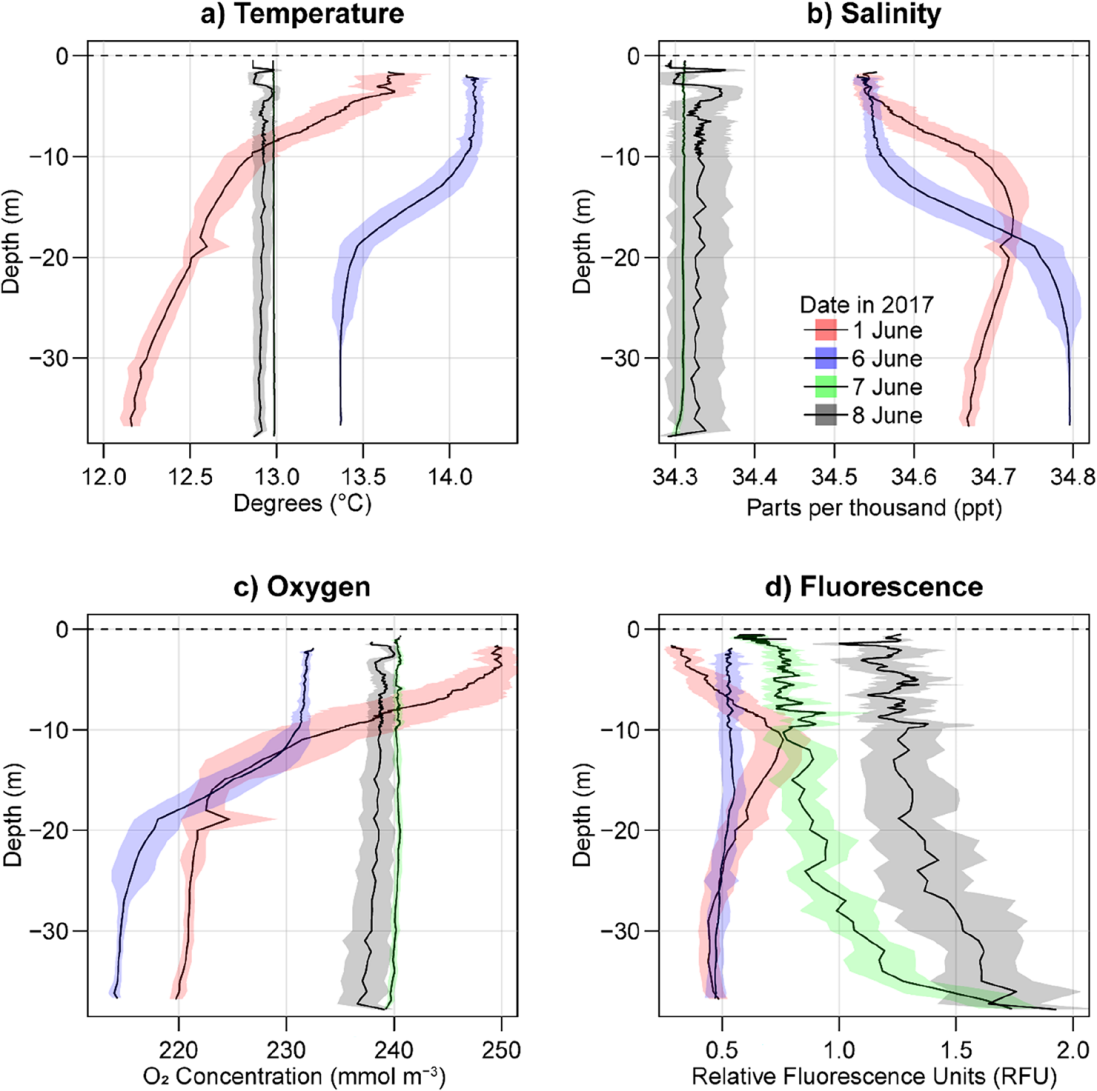
Water column profiles representing temperature, salinity, oxygen and fluorescence, during calm conditions (1 and 6 June), in the middle of the storm (7 June), and after a storm (8 June) in 2017.

Water column temperature profiles taken on 1 June 2017 (n = 23) and on the morning of 6 June 2017 (n = 29) displayed stratified conditions with surface temperatures averaging between 5 to 12% higher than bottom temperatures (Fig. 3a). Temperature profiles taken on 7 (n = 3) and 8 June 2017 (n = 6) were homogenous with similar values in surface and bottom temperatures (12.9°C average). On 1 June and the morning of 6 June, CTD profiles showed increasing salinity and decreasing oxygen in greater depths (Fig. 3b-c). Water column profiles taken on 7 and 8 June 2017 displayed homogenous depth distributions for salinity and oxygen. showing reduced values for salinity (34.24–34.37 ppt) and enhanced oxygen concentrations (239 mmol m^−3^) compared to mean values taken before the storm (pre-storm salinity = 34.67 ppt, pre-storm oxygen = 225 mmol m^−3^; Fig. 3). Fluorescence profiles taken on 1 June 2017 showed a subsurface peak at 10 m which was lost after the storm. Profiles from 7 and 8 June 2017 featured a conspicuous increase in fluorescence throughout the water column compared to the previous measurements (Fig. 3).

### Inorganic nutrients

**Figure 4:**
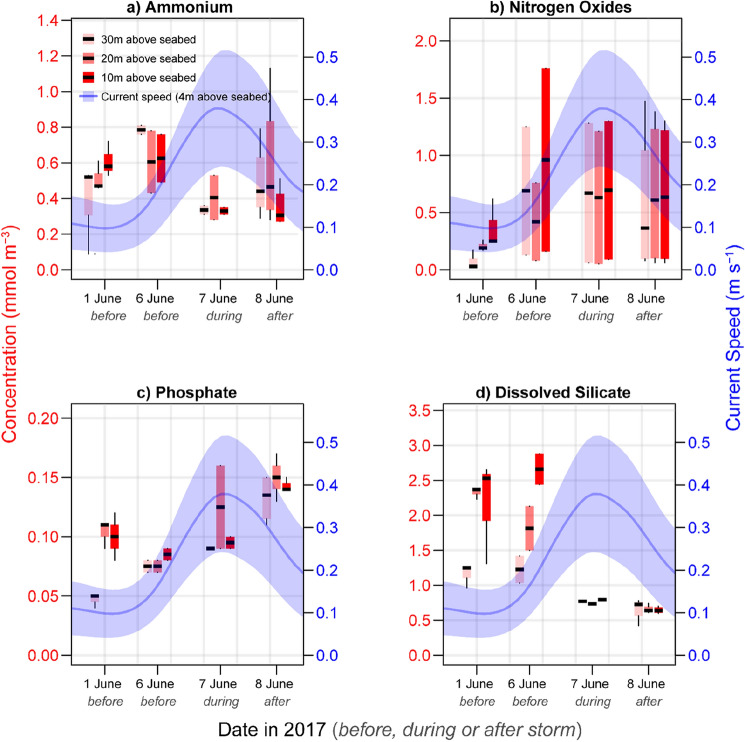
Concentrations ammonium (NH_4_^+^), nitrogen oxides (NO_X_^−^), phosphates (PO_4_^3−^) and dissolved silicates (DSi) measured at 10 (dark red), 20 (medium red), and 30 (light red) meters above the seabed from 4 different dates. Dates 1 and 6 June 2017 represent calm conditions while 7 and 8 June 2017 were taken during and after a summer storm.

From niskin bottles mounted on the CTD frame, water samples were taken at three depths (10, 20, and 30 m above the seabed) on June 1st, June 6th and on June 7th and 8th. On June 1st (5 days before the storm) the concentrations for ammonium (NH_4_^+^), nitrogen oxides (nitrate + nitrite = NO_x_), phosphates (PO_4_^3−^), and dissolved silica (DSi) showed higher concentrations closer to the seabed, typical for stratified conditions (Fig. 4). Average levels of NO_x_ and variability between samples were elevated between 6 and 8 June 2017 (mean ± sd; 0.65 ± 0.61 mmol m^−3^) compared to samples taken on 1 June 2017 (0.21 ± 0.17 mmol m^−3^). DSi concentrations on 6 June 2017 remained lower close to the water surface (30 m above seabed) while samples taken on 7 and 8 June 2017 displayed conspicuously low and homogenous concentrations (0.69 ± 0.094 mmol m^−3^) compared to samples taken between 1 and 6 June 2017 (1.9 ± 0.66 mmol m^−3^). Phosphates displayed increased concentrations at all depths on 8 June 2017 (0.14 ± 0.016 mmol m^−3^) compared to all other dates (0.097 ± 0.033 mmol m^−3^).

### Particle and oxygen dynamics

**Figure 5:**
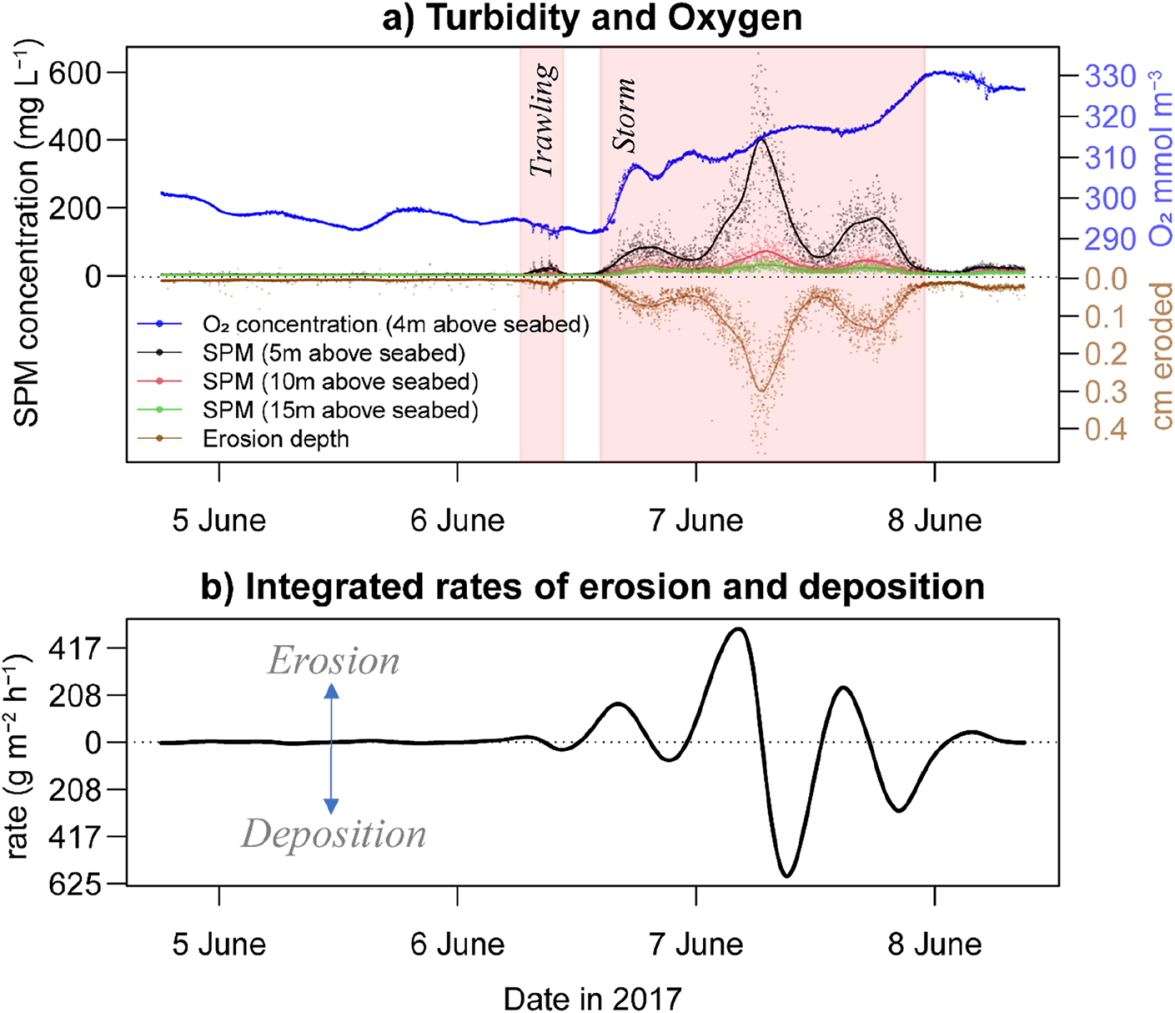
Time series of suspended particulate matter (SPM) concentration and dissolved oxygen content and the calculated depth of resuspended sediment required to support the water column SPM load (**a**) and estimated erosion/deposition rates (**b**). The storm period in this study was defined as the time period when the water currents surpassed 0.35 m s^−1^ which exceeds the maximum tidal currents observed in the five days measured before the 6 June 2017.

Figure 5 describes the progression of oxygen and turbidity in the water column from 5 to 8 June with the calculated depth of seabed erosion needed to sustain the observed SPM (suspended particulate matter) concentrations as well as the integrated erosion and deposition rates. This data overlaps with the sediment resuspension observed during a local trawling event^7^ as well as the time period with elevated currents that distinguishes the ‘storm period’ featured in the current study. Turbidity during 6–7 June 2017 displayed three conspicuous peaks and reached its maximum (620 mg SPM L^−1^) in the early evening of 7 June 2017 (Fig. 5a).

The depth of seabed erosion needed to sustain the SPM concentrations was estimated after integrating the water column sediment load measured from 5, 10, and 15 m above the seabed. Dividing the integrated SPM (g m^−2^) with sediment bulk density measurements (g cm^−3^) allows an for estimation of the eroded seabed (cm) within the resuspended sediments in the water column. The estimated erosion depth reached as high as 0.7 cm during peak turbidity, however, average values during this period measured approximately 0.3 cm (Fig. 5a). The first-derivative of the time series featuring integrated SPM concentrations (Supplementary Fig. S1) gives the estimated erosion and deposition rates (Fig. 5b). The peak in the sediment erosion rate (501 g m^−2^ h^−1^) was followed by a rapid shift towards sediment deposition which reached a maximum of 592 g m^−2^ h^−1^ (Fig. 5b).

On 6 June 2017, oxygen measurements taken at 4 m above the seabed displayed abrupt reductions corresponding directly with turbidity peaks created during experimental bottom trawling (Fig. 5a). Further details on this specific effect are documented in a Frisian Front trawling study^7^. At approximately 14:30 CEST on 6 June 2017, a sharp increase in near-bed oxygen from 293 to 308 mmol m^−3^ was observed within a 2 h period (Fig. 5a). This progressed more gradually to 331 mmol m^−3^ by the evening of 7 June 2017. Temperature and salinity exhibited a gradual decline (13.75 °C–12.86 °C ; 34.94 to 34.35 salinity) from 6 to 8 June 2017 (Supplementary Fig. S2 and S3). Before the onset of the storm, water column temperatures fluctuated consistently between mixed and more stratified conditions in accordance to the tidal cycle (Supplementary Fig. S2).

## Discussion

The study encountered several limitations due to unforeseen circumstances such as the absence of continuous water current and trajectory data prior to the storm event. Disruptions caused by this specific storm resulted in the unbalanced experimental design documented in bottom trawl experiments in the same area^33^. Incidentally, much of the data in the current study was only recorded when it became unsafe for the research vessel to retrieve the *in-situ* measurement devices until the inclement weather had passed. Originally intended to record only trawling impacts, these devices were able to capture insights on physico-chemical changes occurring before and during periods of natural high disturbance. This unique opportunity allowed for a direct assessment of both natural and anthropogenic bottom disturbances within the same area and timeframe.

It is possible that our results reflect a cumulative impact of a storm and prior trawling activities. Nevertheless, bottom current speeds exceeding 5 km per hour during the storm, suggest that any cumulative effect would occur early in the measurement period. Water column effects from the trawling experiments, conducted between 100 and 400 m away from the measurement site, are likely to have been advected well beyond the measurement station before the midpoint of the storm period.

Our findings provide evidence for the hypothesis that storms export large amounts of sediment from the area to northward regions, potentially limiting annual sediment accumulation in the Frisian Front^30,32^. Furthermore, the analysis of water and air current trajectories enhances our comprehension of the physical processes that precede the natural resuspension of southern North Sea sediments.

For approximately 2 h, strong southerly (originating from the south, blowing towards the north) wind gusts occurred 8 h prior to the onset of elevated bottom water currents (> 0.35 m s^−1^). Following this relatively short windy period, the enhanced water currents persisted for a minimum of 41 h continuing until the final measurement recorded at the end of our study (Fig. 1 and 2). The storm event demonstrated noticeable directional variations in both water and air trajectories, with water moving from southwest to northeast and the air shifting from south to north. These observations highlight the influence of the Coriolis effect on the movement of air and water masses.

Residual currents in the North Sea transport southern water masses into the Frisian Front which decelerate upon reaching greater depths^29^. The water masses, located directly to the south, typically exhibit more homogenized water column conditions and lower salinities^34^. During the calm period preceding the storm, water temperatures fluctuated between mixed and stratified conditions (Supplementary Fig. S2) indicating the influence of back-and-forth tidal currents that bring different water masses into the Frisian Front, along with small-scale daily resuspension events (Supplementary Fig. S6). This region experiences both stratified and mixed conditions^35^ allowing for the daily advection of these conditions past the measurement location.

The storm-enhanced transport and mixing of water masses is evident in the decreased salinities and homogenous water column profiles observed from before and after the storm (Fig. 3 and Supplementary Fig. S3), a pattern aligning with observations of wave induced mixing in Dutch and German coastal zones^36^. Salinity measured during and after the storm consistently registered lower values compared to any point measured before the storm (Fig. 3) indicating the influx of fresher water from the southwest into the Frisian Front. Although the absolute change in salinity appears relatively minor compared to observations of typhoon-related impacts in coastal waters^10,22^, this result, coupled with our estimations for storm-induced particle transport (Fig. 2), suggests a potentially higher influence from terrestrial sources on post-storm biogeochemistry in the Frisian Front.

The displacement of water masses in the sampling area coincided with a reduction of dissolved silicates (DSi) and a gradual increase in phosphates (PO_4_^3−^) in the water column (Fig. 4). Enhanced fluorescence measurements after the storm suggest higher levels of chlorophyll-a and the potentially increased biological uptake of DSi by diatoms^37^. Increased primary production and mineralization of organic matter are well-documented effects of storm disturbances^11,19–21,23,27^. The storm-induced release of phosphate, possibly through heightened mineralization or nutrient desorption from sediment particles presents potential mechanisms influencing the altered biogeochemical conditions^18^. Regardless of the specific mechanism, it is probable that differing biogeochemical characteristics observed after the storm originated from water masses transported from the southwest, potentially reflecting terrestrial sources of nutrients and OM from more coastal waters.

Within a span of 3.4 h the maximum erosion rate shifted towards peak deposition, driven by a change in tidal current direction and a subsequent decrease in current velocity (Fig. 5). Storm events are known to trigger episodic pulses of deposition and erosion^38^. Erodible conditions at the study site may have been further intensified by recent trawling destabilizing some of the upper sediment of the seafloor. Five days prior to the storm event, layers of deposited ‘fluffy’ fine sediment were observed and were attributed to experimental trawling conducted in the Frisian Front^33^.

The concentration of SPM (suspended particulate matter) recorded during the storm was up to ten times higher than what was measured during bottom trawling (Fig. 5a), aligning with similar comparisons of storm versus trawl mediated turbidity^39^. Contrary to trawl-induced sediment resuspension, which caused intermittent decreases in bottom O_2_ concentrations^7^, near-bed oxygen concentrations during^7^ the storm led to a drastic and sustained increase in oxygen levels which persisted during the period of heightened currents (Fig. 5a). This outcome appears to diverge from several studies alluding to oxygen consuming decomposition of OM as the dominant biogeochemical process occurring after a storm event^11,19–21,25–27^. However, our study, unlike those post-storm assessments, provides insights into dynamic processes occurring amidst stormy conditions. While it is still plausible for oxygen-consuming processes to dominate in the days following the storm, our observations suggest inherent differences in how trawl and storm induced disturbances can directly oxidize reduced substances. A closer examination of the depth of sediment penetration resulting from trawling and storms as well as the role of wave mixing, can offer insight on the different mechanisms underlying storm and trawl induced impacts.

Bottom trawling in the Frisian Front has been reported to mobilize the upper 0.7–0.8 cm of sediment (erosion) while penetrating and mixing 1–3.4 cm underneath this layer^40^. Our study estimated that during the height of the storm, the average amount of SPM was equivalent to a 0.3 cm layer of resuspended sediments with a maximum depth observed at 0.7 cm (Fig. 5a). This is comparable to a study conducted on the UK coast of the North Sea, where a storm induced a maximum erosion depth of 1 cm at a water depth 25 m, with average erosion depths values falling an order of magnitude lower^41^. We hypothesize that trawling, with its ability to resuspend and reoxidize substances from several centimetres within the seafloor^7,40^, releases higher concentrations of reduced compounds per square meter compared to short-term storm perturbations. This causes immediate reductions in dissolved oxygen and an elevated presence of degraded OM in suspension. This is supported by a recent study in the Mediterranean which attributes higher levels of degraded OM being resuspended and transported by trawling activities compared to storm events^17^. Nevertheless, the capacity for storms to transport fresh OM and nutrients into a system can trigger longer-term changes to dissolved oxygen levels. Following the initial increase of oxygen from wave mixing, local biogeochemistry may be stimulated by the storm-induced arrival of fresh OM^11,20,25^ leading to reductions in dissolved oxygen ^21,26,27^. Coastal areas with high anthropogenic influence are particularly vulnerable to storm-induced hypoxia after an initial spike in primary production^19,20^.

Our findings highlight the distinction in the scale of impact between storms and trawling on marine biogeochemistry. While the effects from sediment resuspension from trawling are more localized and straightforward to discern^7,42^, storm-induced alterations on parameters such as oxygen, nutrients and OM, extend over a regional scale, likely stemming from sources several kilometres away from a single measurement point. Deciphering whether the mechanism of these changes originates from sediment resuspension or other contributing factors presents a challenge for researchers. Nonetheless, this study emphasizes the powerful role of storms in the resuspension and transport of sediment and dissolved compounds, a key factor shaping particle distribution and biogeochemical characteristics in continental shelf habitats.

## Methods

### Site description

Measurements were taken in the area of the North Sea known as the Frisian Front (53.7°N, 4.4°E; ~ 34 m depth) which is located ~ 60 km northwest of the Dutch coast and is characterized by muddy sand (Fig. 1c; ^29^). Experimental trawling was conducted on the 1st and the morning of the 6th of June 2017^7^. Enhanced waves and water velocities (> 0.3 m s^−1^) commenced on the early afternoon of 6 June 2017 and continued after data collection ceased on 8 June 2017 (Fig. 2a).

### Data collection

All measuring equipment was deployed from the research vessel (RV) “Pelagia” (Royal NIOZ). A benthic Autonomous Lander for Biological Experiments (ALBEX;^43^) was deployed on 1 and 3 June 2017 in calm conditions as well as the period between 6 and 8 June 2017 in stormy conditions. The lander was equipped with an Acoustic Doppler Velocity meter (ADV; Aquadopp, Nortek) fixed at 1.40 m above the seabed which recorded information on current speed and direction. To measure water column SPM, a mooring, which held optical backscatter point sensors (OBS) at 5, 10 and 15 m above the seabed, was deployed from the RV between 4 and 8 June 2017. The mooring was also fitted with an Aanderaa “SeaGuard” recorder suspended at 4 m above the seabed, which collected information on oxygen concentrations, and water current speeds and trajectories.

A conductivity temperature and depth (CTD; Sea-Bird Scientific) recorder which included sensors for dissolved oxygen, and fluorescence was deployed on 1 June 2017 (n = 23), 6 June (n = 29), 7 June (n = 3) and 8 June 2017 (n = 6). More CTD casts were taken on 1 and 6 June and on 7 or 8 June due to the original attempt to capture the effects of the experimental trawling disturbances (using the termed ‘yo-yo CTD’ method). Niskin bottles fixed onto the CTD rosette frame collected water samples from 5, 15, and 25 m depths during each measurement period (3 samples per depth on 1 June; 2 samples per depth on 6 and 7 June, 4 samples per depth on 8 June). Water samples were run through a 0.45 μm mesh filter and stored in 10 mL vials at− 20 ◦C prior to analysis.

### Analytical methods

A SEAL QuAAtro segmented flow analyser^44^ was used to analyse inorganic nutrient concentrations from water samples after the samples thawing. Sediment samples were collected and analysed using the methods outlined in two Frisian Front trawling studies^7,33^.

To estimate the particle paths in respect to distance in calm (1 and 3 June 2017) and stormy (6–7 June 2017) conditions, a simple model was constructed by using the velocity of a fluid particle as the time derivative of the trajectory. Erosion and deposition rates were obtained after integrating the SPM concentrations (g m^−3^) measured by the OBS sensors over the entire water column and then fitting a cubic smoothing spline through the time series to obtain the average values (Supplementary Fig. S1). The first-derivative of the integrated SPM concentrations (g m^−2^) was used as the estimations of erosion for positive values and deposition for negative values (g m^−2^ h^−1^; Fig. 5b). To estimate the depth of eroded seabed needed to sustain the amounts of resuspended sediment, values for integrated SPM (g m^−2^) were divided by the bulk density of seafloor sediment (g cm^-3^) to estimate the thickness of the eroded layer (cm). A cubic smoothing spline was fitted through the erosion depth time series visualize the average erosion depths over time (Fig. 5a).

All calculations were performed in R^45^.

### Supplementary Information

Supplementary Information.

## Data Availability

The datasets used in this study can be made available upon request to the corresponding author.
