# Supplementary material for: Biogeochemical dynamics in a marine storm demonstrates differences between natural and anthropogenic impacts
Source: Sci Rep. 2024 Apr 16;14:8802. doi: 10.1038/s41598-024-59317-8 (PMC11021396; doi:10.1038/s41598-024-59317-8)
Supplement: Supplementary file 1 — Supplementary Information. [file 41598_2024_59317_MOESM1_ESM.pdf]

Supplementary Material for :

Biogeochemical dynamics in a marine storm demonstrates differences between natural and anthropogenic impacts

*Justin Tiano[ab], Rob Witbaard[b], Theo Gerkema[b], Karline Soetaert[b]

[a]*Wageningen Marine Research, Wageningen University & Research, The Netherlands*.

[b]*Royal Netherlands Institute for Sea Research (NIOZ) and Utrecht University, Department of Estuarine and Delta Systems, The Netherlands*

*Corresponding author: Justin C. Tiano (justin.tiano@wur.nl)

Number of pages: 7

Number of figures: 6

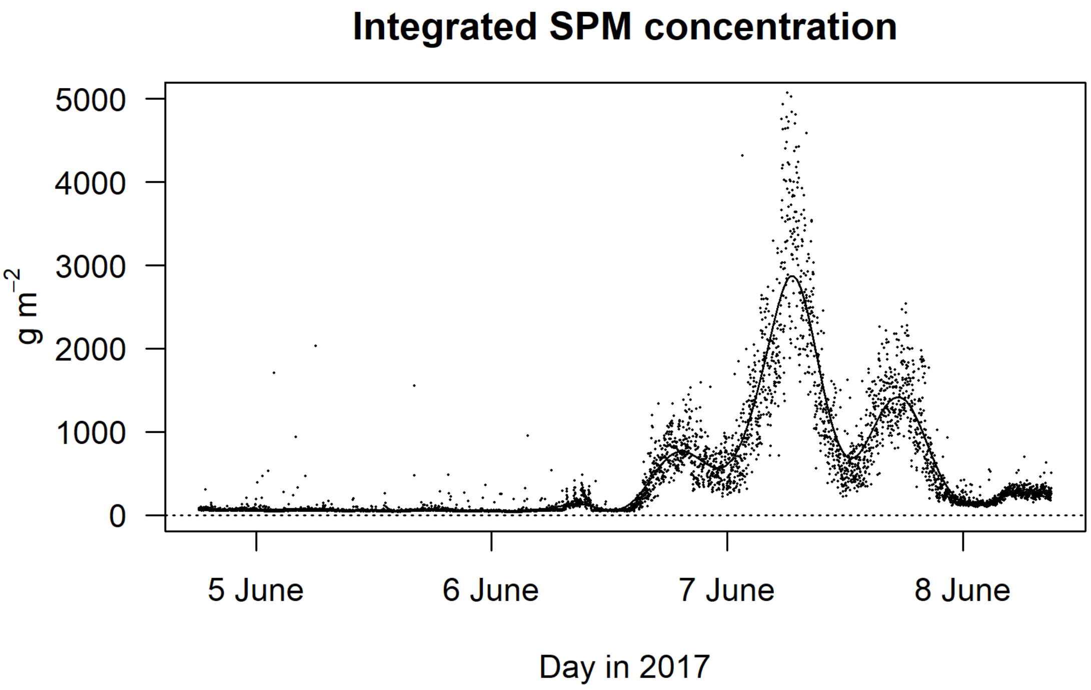

Fig. S1. Time series showing the integrated suspended particulate matter (SPM) concentration over time measured from 5, 10, and 15 m above the seabed.

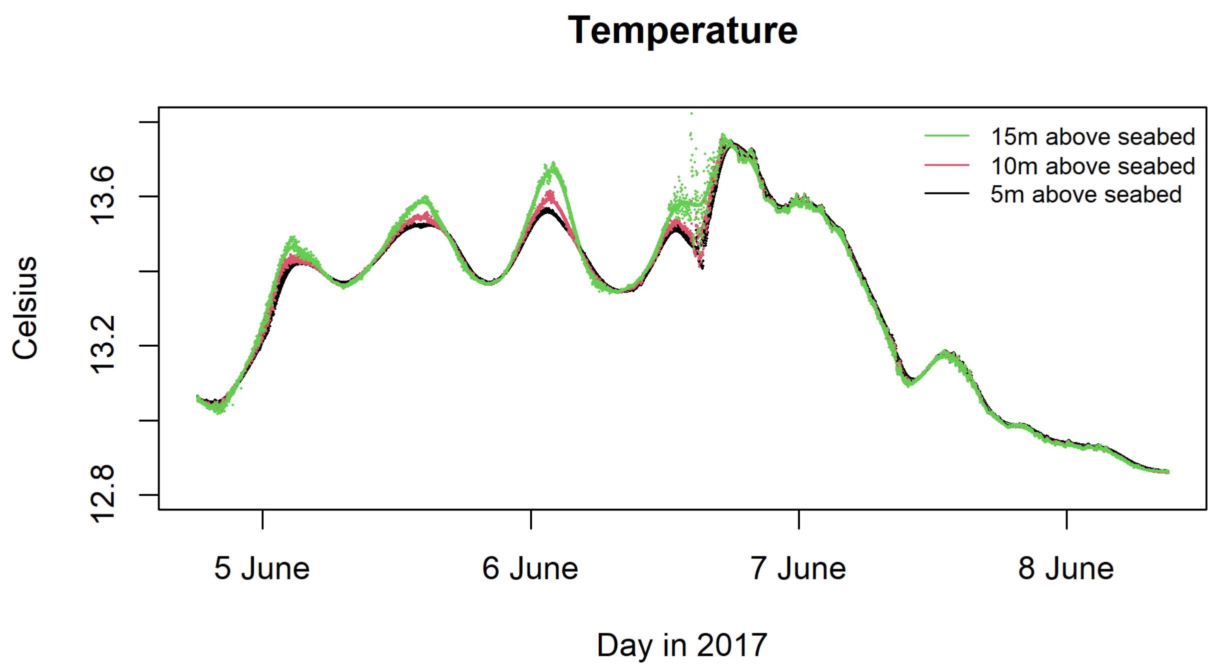

Fig. S2. Time series of temperature from 5, 10, and 15 m above the seabed measured from 5 – 8 June 2017 in the Frisian Front.

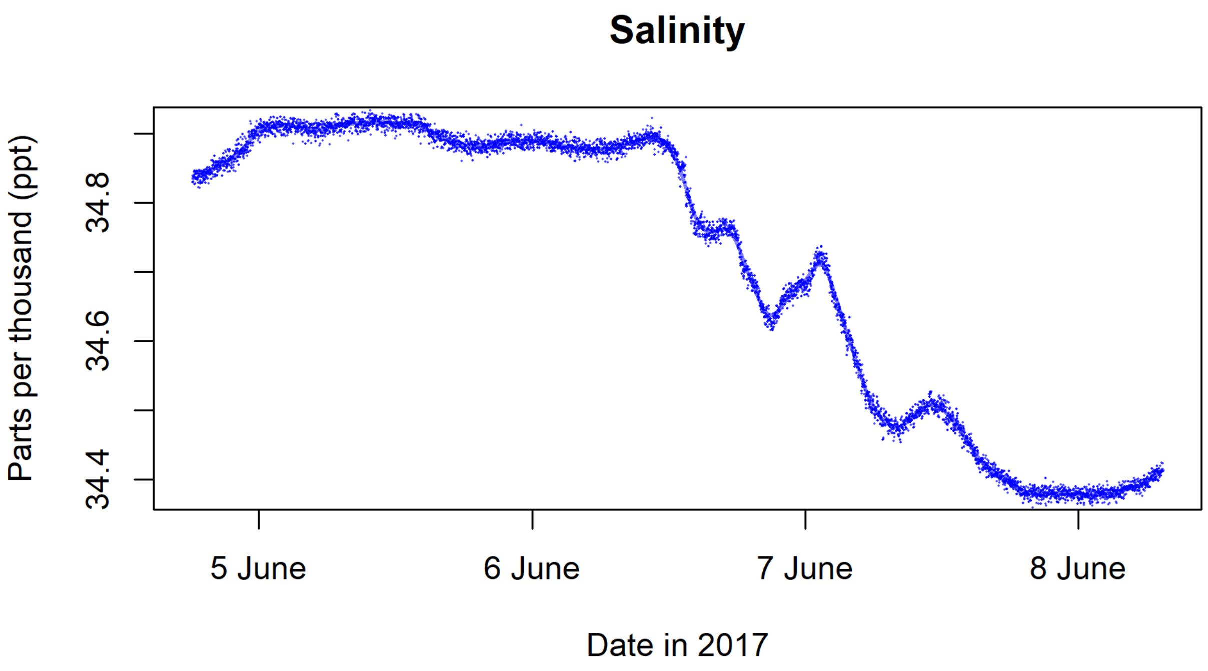

Fig. S3. Time series of salinity from 4 m above the seabed measured from 5 – 8 June 2017 in the Frisian Front.

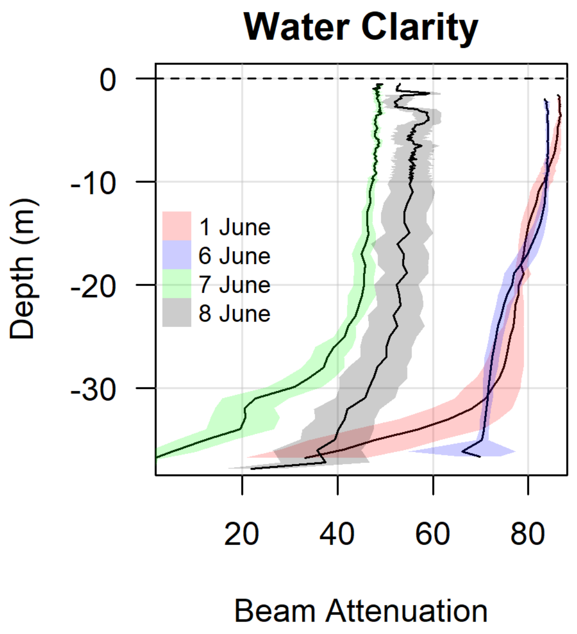

Fig. S4. Water column profiles representing beam attenuation, during calm conditions (1 and 6 June), in the middle of the storm (7 June), and after a storm (8 June) in 2017.

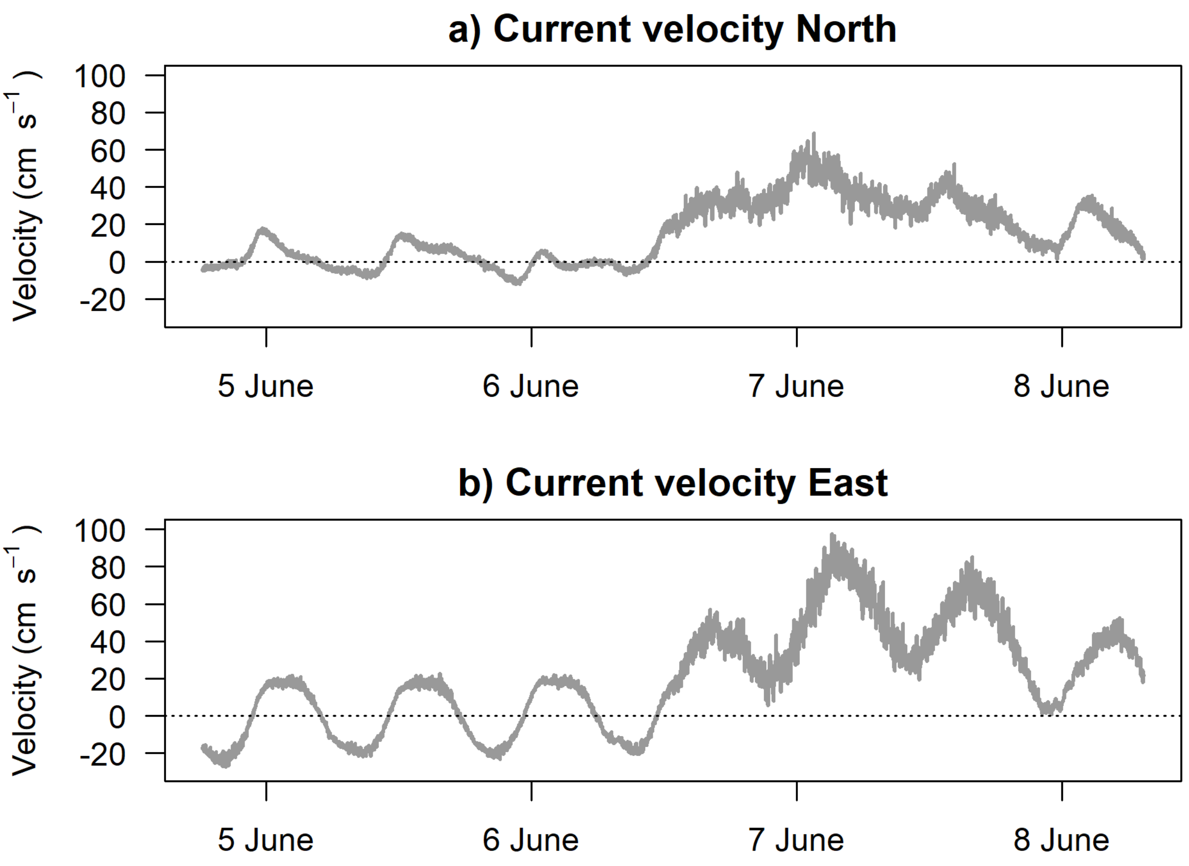

Fig. S5. Water current velocities measured from 4 meters above the seafloor for Northward (a) and Eastward (b) trajectories.

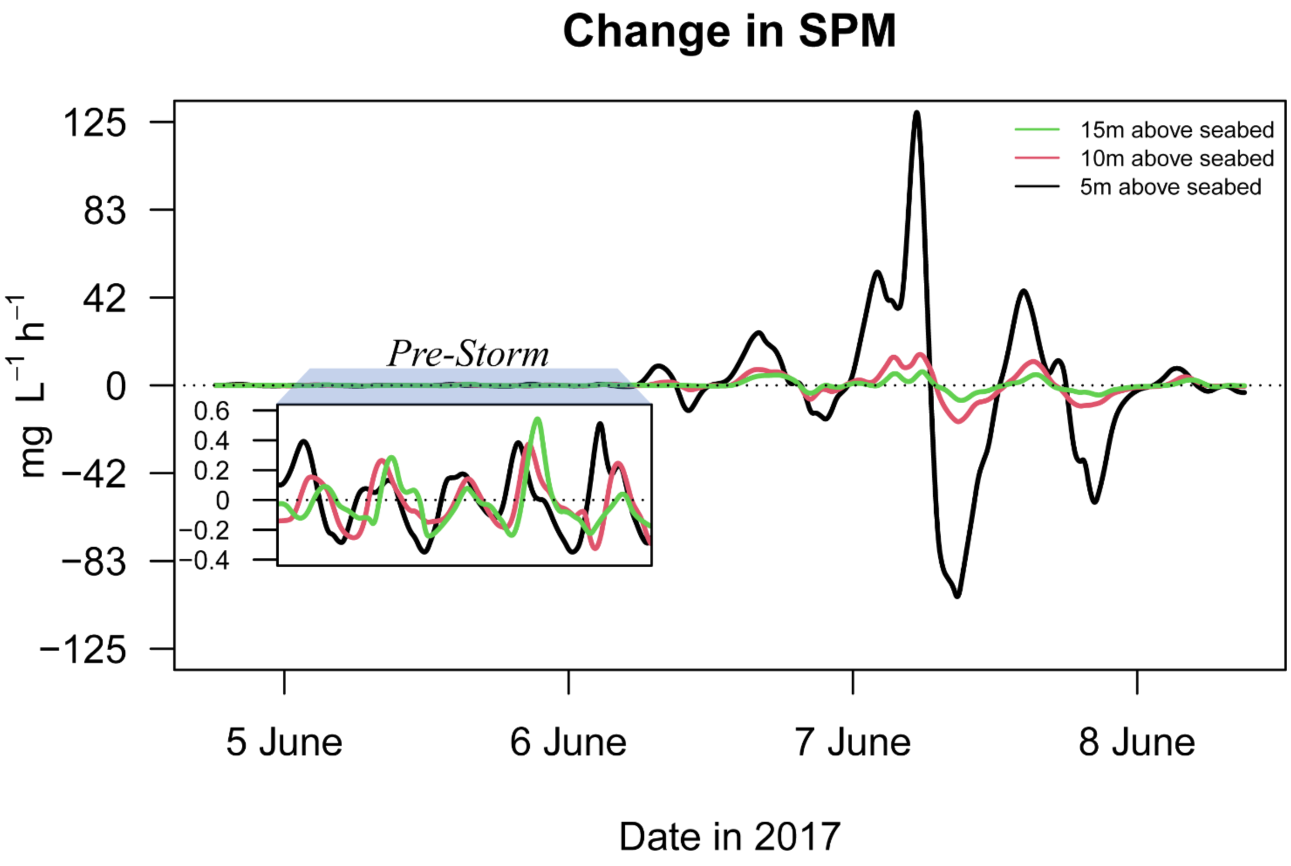

Fig. S6. Time series showing the rate of change in suspended particulate matter (SPM) concentration measured from 5, 10, and 15 m above the seabed. Values represent the first-derivative of the cubic smoothing spline fitted onto the SPM concentration data.
